# Clinical Characteristics of Candidemia Due to *Candida parapsilosis* with Serial Episodes: Insights from 5-Year Data Collection at a Tertiary Hospital in Korea

**DOI:** 10.3390/jof10090624

**Published:** 2024-09-01

**Authors:** Eun Jeong Won, Heungsup Sung, Mi-Na Kim

**Affiliations:** Department of Laboratory Medicine, Asan Medical Center, University of Ulsan College of Medicine, Seoul 05505, Republic of Korea; sung@amc.seoul.kr (H.S.); mnkim@amc.seoul.kr (M.-N.K.)

**Keywords:** *Candida parapsilosis*, persistent candidemia, clinical characteristics, echinocandin

## Abstract

*Candida parapsilosis* is a common cause of non-*albicans Candida* species causing candidemia, particularly invasive candidiasis. This study aimed to characterize candidemia due to the *C. parapsilosis* complex with serial episodes, including clinical and mycological features. Methods: Blood isolates of the *C. parapsilosis* complex were collected from February 2019 to January 2023 at a tertiary Korean hospital. Species identification was performed using Vitek 2 or matrix-assisted laser desorption/ionization time-of-flight mass spectrometry, and antifungal susceptibility testing was performed using the Sensititre YeastOne^®^ system. Clinical information was collected, and characteristics were analyzed according to single or serial isolates. Results: A total of 586 blood isolates of the *C. parapsilosis* complex were recovered from 68 candidemia patients during the study period. Of them, only the first isolate per patient was investigated. The only two isolates were resistant to fluconazole and no isolate was resistant to echinocandins, amphotericin B, or 5-FC. A single episode of candidemia occurred in 35 patients, while serial episodes occurred in 33 patients. Underlying liver diseases, use of vasopressors, ICU admission, severe sepsis, and CVC use were more frequent in patients with serial episodes. There was no significant difference in the median MIC values of antifungal agents or the use of azoles or amphotericin B between single and serial episodes. However, patients with serial episodes more frequently received echinocandin therapy. Overall, there was no significant difference in the 30-day mortality rate between patients with single and serial episodes. Conclusion: Our data indicate that several factors related to the underlying conditions of the patients are associated with *C. parapsilosis* candidemia with serial episodes, rather than the characteristics of *Candida* itself.

## 1. Introduction

*Candida* bloodstream infections (BSIs) are the most common nosocomial fungal infections and are associated with high rates of mortality, especially among immunocompromised patients such as those in intensive care units (ICUs) [1]. Although *Candida albicans* remains the most prevalent species, there has been a global shift toward non-albicans *Candida* (NAC) species [2]. Among these, *Candida parapsilosis* (*C. parapsilosis*) ranks as the second to fourth most common species causing candidemia, depending on patient age and geographic location [3]. It is the second most isolated species in Latin America or southern European countries [4,5,6,7], and it even outranks *C. albicans* infections in some European, Asian, and South American hospitals [8,9,10]. On the other hand, it is reported as the third and fourth most common causative species of candidemia in many other areas of the world [11]. In Korea, *C. parapsilosis* has been identified as the fourth most common cause of candidemia, either in ICU or non-ICU settings [12]. Although *C. parapsilosis* is typically considered susceptible to azole antifungals, recent reports indicate an increasing incidence of fluconazole-resistant (FR) *C. parapsilosis* BSIs in several countries [13,14,15]. This resistance poses a significant clinical challenge, since fluconazole is commonly used to treat *C. parapsilosis* BSIs [16]. The clonal transmission of FR C. *parapsilosis* among hospitalized patients further exacerbates the issue. The main mechanism related to fluconazole resistance is the presence of ERG11p substitutions, dominated by Y132F amino acid substitution. In Korea, the emergence and nosocomial spread of *C. parapsilosis* Y132F isolates, which exhibit a higher propensity for clonal transmission and persistence in hospitals compared with other fluconazole-resistant strains, has been documented [17]. Notably, during the COVID-19 pandemic, the incidence of *C. parapsilosis* complex (CPC) candidemia increased by 1.6-fold compared with the pre-pandemic period, a much higher rate than observed for other *Candida* species [18]. In addition, in that study, CPC candidemia was two times more persistent than that caused by other common *Candida* species, and this persistence was strengthened during the pandemic [18]. To understand the reasons behind this increase, we conducted this study to investigate the clinical characteristics of CPC candidemia with serial episodes, alongside molecular epidemiological analyses.

## 2. Materials and Methods

Blood isolates of CPC were collected from January 2019 to December 2023 at a tertiary Korean hospital which had 2732 beds, including 170 intensive care unit beds, and had annually 3,469,589 outpatients, 926,794 inpatients, and performed 70,892 highly sophisticated surgeries per year. Only the first isolate per patient was included in the study. Species identification was performed using the Vitek 2 yeast identification card (Vitek 2 Yeast ID; bioMérieux, Marcy l’Etoile, France) according to the manufacturer’s instructions, or using matrix-assisted laser desorption/ionization time-of-flight mass spectrometry (Bruker Biotyper; Bruker Daltonics GmbH, Bremen, Germany) [18,19]. Antifungal MICs for fluconazole, voriconazole, posaconazole, itraconazole, anidulafungin, caspofungin, and micafungin were determined using the Sensititre YeastOne^®^ system (Thermo Fisher Scientific, Waltham, MA, USA), which is a commercial colorimetric test for the determination of minimum inhibitory concentrations based on the broth microdilution. MIC interpretive criteria were based on species-specific Clinical and Laboratory Standards Institute (CLSI) clinical breakpoints according to the guidelines in the CLSI document M60 ED1 [20]. DNA was extracted using a QIAamp DNA Mini Kit (QIAGEN, Hilden, Germany) according to the manufacturer’s instructions. The *ERG11* gene was amplified and sequenced to fluconazole-resistant isolates as previously described [17,21]. Two sets of primers used for the amplification and sequencing of *ERG11* were as follows: 5′-CGAGATAATCATCAACGAACATTC-3′ (CP-1F) and 5′-CGTTTAAAACATCCAAAGACCTTA-3′ (CP-1R), 5′-AATCTGAGGGTTTCCTTGATGGT-3′ (CP-2F) and 5′-AAAGACCGCATTGACTACCGAT-3′ (CP-2R), respectively. Amplification conditions were as follows: an initial denaturation step for 1 min at 94 °C, followed by 30 cycles of denaturation for 30 s at 94 °C, annealing for 60 s at 55 °C, elongation for 90 s at 72 °C, and a final elongation step for 10 min at 72 °C. Polymerase chain reaction (PCR) products were purified using a PCR purification kit (GeneAll Biotechnology, Seoul, Repuloic of Korea) according to the manufacturer’s recommendations. The PCR products were sent to Cosmogenetech (Seoul, Repuloic of Korea) for direct sequencing using the same PCR primer pairs. Nucleotide sequences were compared with the available corresponding sequence of *C. parapsilosis* ATCC 22019 (GenBank accession no. GQ302972). The genetic relatedness of fluconazole-resistant isolates was further investigated by a combination of the microsatellite and minisatellite markers (CP1, CP4, CP6, and STR-ACA) according to previous reports [17,22,23]. Clinical information, including demographics, clinical status at the time of positive culture, therapy-related factors, and outcome of fungemia, was collected retrospectively through medical records. Clinical status at positive culture was assessed within 30 days before the occurrence of candidemia and included factors such as prior surgery, total parenteral nutrition use, prior antifungal exposure, immunosuppressive therapy, and neutropenia (defined as an absolute neutrophil count of 500/mm^3^). Patient outcome (survival or death) was assessed as all-cause mortality at 30 days after the first positive blood culture result. In this study, we defined persistent candidemia with serial isolates when the same isolates were serially from a positive blood culture for ≥3 days after the initiation of antifungal therapy [18,24]. This study was approved by the Institutional Review Board (IRB) of the Asan Medical Center (approval no. 2023-0467). The IRB waived the requirement for informed consent due to the retrospective nature of the study.

### Statistical Analysis

Quantitative variables are expressed as median and interquartile ranges, while categorical variables are presented as counts and percentages. Categorical variables were compared between groups using the χ2 or Fisher’s exact test. Continuous variables were compared between groups using the independent *t*-test for normally distributed data and the Mann–Whitney U test for non-normally distributed data. Statistical analysis was performed using Prism 5.0 (GraphPad Software in San Diego, CA, USA). A *p*-value of less than 0.05 was considered statistically significant.

## 3. Results

During the study period, a total of 586 blood isolates of CPC were recovered from 68 candidemia patients. The first isolates of each patient were only assessed and their MIC_50_ and MIC_90_ values (Table 1). In this study, no isolates were resistant to echinocandins, amphotericin B, or 5-FC. The only two isolates were resistant to fluconazole and harbored *ERG11* mutations [Y132F (n = 1), K143R (n = 1)].

Table 2 describes mycological and clinical characteristics of fluconazole-resistant isolates found in this study. One isolate harboring Y132F caused a single episode of candidemia. The patient did not use CVC and showed a fair response without any antifungal therapy. The other fluconazole-resistant isolate which harbored K143R was recovered from a patient who was diagnosed with infective endocarditis with serial episodes of candidemia. The patient previously received anidulafungin therapy for 5 days, and breakthrough occurred. After then, amphotericin B was administered and *C. parapsilosis* disappeared after a week. Upon the analysis of the combination of microsatellite and minisatellite markers, these two fluconazole-resistant isolates were not closely related to each other genetically.

Table 3 summarizes clinical and mycological characteristics of CPC candidemia patients according to the number of episodes. Single episodes of candidemia occurred in 35 patients, while serial episodes occurred in 33 patients. The frequencies of underlying liver diseases (serial episodes vs. single episode; 27.3% vs. 2.9%, *p* = 0.004), use of vasopressors (33.3% vs. 11.4%, *p* = 0.029), ICU admission (45.5% vs. 22.9%, *p* = 0.049), presence of severe sepsis (45.5% vs. 20.0%, *p* = 0.024), and central venous catheter (CVC) use (78.8% vs. 45.7%, *p* = 0.005) were higher in the patients with serial episodes compared with those with a single episode (Table 3). There was no significant difference in the strategy of CVC removal between them or the duration of CVC (median duration with CVC was 3 days in both). There was no significant difference in the median MIC values of antifungal agents between single and serial isolates. Similarly, there was no significant difference in the use of azoles or amphotericin B between patients with single or serial episodes. However, patients with serial episodes of candidemia were more frequently treated with echinocandins compared with those with a single isolate (patients with serial episodes vs. a single episode; 72.7% vs. 48.6%, *p* = 0.041). Overall, there was no significant difference in the 30-day mortality rate of candidemia patients according to the number of episodes.

## 4. Discussion

Among the NAC species, *C. parapsilosis* has emerged as an important causative agent of invasive candidiasis over the past decade, despite having been considered susceptible to triazoles [25,26,27]. In Korea, the prevalence of *C. parapsilosis* has fluctuated over time. According to the Korean Global Antimicrobial Resistance Surveillance System (Kor-GLASS), *C. glabrata* and *C. tropicalis* rose to become the second and third most common causes of candidemia from 2020 to 2021, surpassing *C. parapsilosis* [28]. In line with the current epidemiology in Korea, we also found that CPC was the fourth most common species, exhibiting 9.5% of candidemia; a total of 798 (average number of candidemia, 159.6 cases per year) candidemia patients were observed during the study period. Recent studies from Europe [29,30], Africa [31], and Latin America [32] have reported high rates of fluconazole and/or azole resistance in *C. parapsilosis*, particularly in strains harboring the Y132F mutation in the *ERG11* gene. These Y132F C. *parapsilosis* isolates have been implicated in hospital outbreaks [14,30,31,33], with some clusters becoming endemic within hospital environments [17,34]. The emergence and clonal spread of fluconazole-resistant *C. parapsilosis* isolates, especially those with the Y132F mutation, pose a serious public health threat worldwide.

In Korea, the clonal spread of fluconazole-resistant *C. parapsilosis* isolates with the Y132F mutation has been documented in only two hospitals (each with >2000 beds) in Seoul [17,28]. Recently, we observed a 1.6-fold increase in the incidence of CPC candidemia during the COVID-19 pandemic at another major center with approximately 3000 beds in Seoul [18]. We speculated that this increase was not caused by the Y132F clone, because the fluconazole resistance rate remained relatively low. During this 5-year period, only two isolates exhibited fluconazole resistance and they harbored two kinds of well-known *ERG11* mutations, respectively. Y132F and K143R *ERG11* substitutions causing alteration in the enzyme’s structure, reducing drug affinity and rendering resistance, has been reported as the major cause of fluconazole resistance [35]. It was previously reported that the strains with the Y132F mutation previously had a higher tendency for clonal transmission and persistence in a hospital environment [36]. In this study, however, one isolate harboring Y132F was not related to persistence or therapeutic challenge, contrasting with previous findings of its high transmissibility and resistance issues. Instead, another fluconazole-resistant isolate which harbored K143R was presenting as breakthrough during anidulafungin therapy. These two cases were not genetically related and exhibited a fair prognosis. However, a Turkish study announced a link between these mutations in genes implicated in mortality and they found that the isolates with Y132F were three times higher than that caused by isolates with Y132F + K143R [29]. The antifungal resistance and mortality of candidemia can differ according to the study cohort or country. In recent Korean data, the overall crude 30-day rate of mortality due to *C. parapsilosis* candidemia was 27.4%, quite lower than the reports from other countries [5,6,23,29,37,38,39]. In that study, the mortality rate of *C. parapsilosis* candidemia was not significantly different according to the presence of Y132F [23]. Moreover, our 5-year data collection showed no significant differences in antifungal susceptibility patterns between single and serial isolates. Differences among various centers could be attributed to infection control policies, prior azole use, the composition of patient populations vulnerable to candidemia, and the spread of fluconazole-resistant clones across hospitals [40].

Further, we focused on the fact that half of the CPC candidemia cases were persistent. Previous randomized clinical trials reported persistent candidemia in 8% to 15% of candidemia patients [41]. It has been known that *C. parapsilosis* infection itself could be an independent risk factor for persistent candidemia [42,43]. In this study, the presence of underlying liver diseases, use of vasopressors, ICU admission, severe sepsis, and CVC use were notable in persistent candidemia cases. Previous abdominal surgery, total parenteral nutrition, fungal colonization, CVC use, broad-spectrum antibiotic use, septic shock, and renal replacement therapy were well-known risk factors for *C parapsilosis* candidemia [42,43,44,45,46]. Among these, CVC is recognized as the most common risk factor. However, when we compared the frequency of CVC removal and the duration of CVC maintenance between single and serial episodes, there was no significant difference. This may be partly supporting the notion that CVC removal is not a definitive solution for managing candidemia.

Notably, liver disease was identified as a risk factor of persistent *C. parapsilosis* candidemia. Patients with liver dysfunction are highly susceptible to *Candida* spp. infections due to impaired phagocytic function, the need for invasive procedures, and exposure to antibiotic treatments [47]. Liver disease may also facilitate candidemia by promoting microbial translocation from the gut, secondary to systemic inflammation, and impairing the local immune system [48]. Our data suggest that the underlying condition of the patient is crucial in the persistence of *C. parapsilosis* candidemia.

There was no significant difference in use of azoles or amphotericin B between candidemia patients with single or serial episodes. However, patients with serial episodes more frequently received echinocandin therapy than those with a single episode. Despite this, candidemia cases with serial episodes did not show significantly worse prognoses, and the resistance rates to azoles or echinocandins were low. *C. parapsilosis* has a naturally occurring amino acid substitution, P660A, in the *FKS1* hot spot 1 region, which may reduce susceptibility to echinocandins in vitro. Previous studies have shown a positive correlation between the incidence of fluconazole-resistant *C. parapsilosis* candidemia and the use of echinocandins for treatment [23,49]. Byun et al. characterized distinct Y132F-sinking isolates of *C. parapsilosis,* partly selected by echinocandin use, and suggested these isolates as indicators of ongoing undetected long-term clonal transfer within hospitals [23]. Although *C. parapsilosis* isolates were susceptible to azoles, their persistent recovery could increase the chance of horizontal transmission through contaminated medical equipment and staff in the clinic, leading to crossover infections between patients. Therefore, it is crucial to diligently monitor antifungal resistance and persistent clones in clinical settings that have experienced fungal infection outbreaks.

Our study had some limitations. Firstly, we focused on clinical analysis for routinely identified clinical isolates; thus, sequencing analysis was not performed for all isolates. Therefore, the *C. parapsilosis* complex could not completely differentiate into *Candida parapsilosis* sensu stricto, *Candida orthopsilosis*, and *Candida metapsilosis*, especially in the case with the identification using the Vitek 2 system [50]. Secondly, the study was based on data from a single center, necessitating cautious interpretation. The findings emphasized that various factors related to the underlying conditions of candidemia patients, rather than the characteristics of the *Candida* itself, were associated with persistent CPC candidemia. Additionally, the presence of underlying liver disease and the use of echinocandin were noted in cases of persistent CPC candidemia. This insight can help in the development of more efficacious antifungal stewardship and treatment protocols to enhance patient outcomes.

## Figures and Tables

**Table 1:** Antifungal susceptibilities of 68 isolates of the *Candida parapsilosis* complex performed using Sensititre YeastOne broth microdilution assay in this study.

Antifungal Agents	MIC (μg/mL)															No. (%) of Isolates *	
	0.01	0.02	0.03	0.06	0.12	0.25	0.5	1	2	4	8	16	MIC Range	MIC_50_	MIC_90_	Resistant	Intermediate
Fluconazole					7	5	23	25	6			2 *	0.12–16	0.5	2	2 (2.9)	0 (0.0)
Voriconazole	21	23	18	4	1		1						0.008–0.5	0.015	0.03	0 (0.0)	1 (1.4)
Posaconazole	9	14	36	5	2	1			1				0.008–2	0.03	0.06	N.A.	N.A.
Caspofungin	7		1	3	6	18	27	6					0.008–1	0.25	0.5	0 (0.0)	0 (0.0)
Micafungin	7	1			2	9	22	25	2				0.008–2	0.5	1	0 (0.0)	0 (0.0)
Anidulafungin		7		1	5	12	27	15	1				0.015–2	0.5	1	0 (0.0)	0 (0.0)
Amphotericin B				1	26	20	19	2					0.06–1	0.25	0.5	N.A.	N.A.
5-FC			2	66									0.03–0.06	0.06	0.06	N.A.	N.A.

* Two isolates resistant to fluconazole harbored *ERG11* mutations. Abbreviations: MIC, minimum inhibitory concentration; N.A., not available; 5-FC, flucytosine.

**Table 2:** Mycological and clinical characteristics of two fluconazole-resistant *Candida parapsilosis* complex bloodstream isolates found in this study.

Isolate	MIC (µg/mL)	*ERG11* *	Microsatellite-Minisatellite Typing **				Clinical Features						
	FLU/VOR/CAS/ANI/MICA		ACA	CP1	CP4	CP6	Candidemia Episode	Age/Sex	Underlying Diagnosis	Previous Antifungal Therapy	Antifungal Therapy	CVC Use	Prognosis at 30-Day
No. 1	16/0.5/0.12/0.06/0.015	Y132F	370/370	243/243	373/373	286/292	Single	73/M	Hypoxic brain injury	None	None	None	Survive
No. 2	16/0.12/0.5/1/1	K143R	247/295	222/222	212/212	187/187	Serial (0, 1, 3, 5, 7)	57/M	Infective endocarditis	ANI (-4~0)	AMB (1~23), VOR (9~156), FLU (24~32)	Yes	Survive

* Non-synonymous substitutions in *ERG11* were found in fluconazole-resistant isolates. ** The numbers before and after the slashes indicate the fragment sizes (bp) of the different alleles obtained with the indicated marker. Abbreviations: FLU, fluconazole; VOR, voriconazole; CAS, caspofungin; ANI, anidulafungin; MICA, micafungin; MIC, minimum inhibitory concentration; CVC, central venous catheter; AMB, amphotericin B.

**Table 3:** Characteristics of candidemia patients caused by the *C. parapsilosis* complex enrolled in this study.

Characteristics		Total	Single Isolate	Serial Isolates	*p*-Value
No. of cases		68	35	33	
Demographics					
	Age, years, median (IQR)	68 (57.75–75)	66 (55–76)	73 (59–75)	
	Male, No. (%)	43 (63.2)	21 (58.3)	22 (66.7)	0.475
Underlying condition					
	Diabetes melitus, No. (%)	10 (14.7)	5 (14.3)	5 (15.2)	0.919
	Liver disease, No. (%)	10 (14.7)	1 (2.9)	9 (27.3)	0.004
	Moderate to severe chronic kidney disease, No. (%)	7 (10.3)	3 (8.6)	4 (12.1)	0.63
	Congestive heart failure, No. (%)	8 (11.8)	3 (8.6)	5 (15.2)	0.399
	Myocardial infarction, No. (%)	11 (16.2)	7 (20)	4 (12.1)	0.377
	Chronic obstructive pulmonary disease, No. (%)	8 (11.8)	3 (8.6)	5 (15.2)	0.399
	Peripheral vascular disease, No. (%)	1 (1.5)	1 (2.9)	0 (0)	N.A.
	Cerebro-vascular disease, No. (%)	10 (14.7)	6 (17.1)	4 (12.1)	0.558
	Dementia, No. (%)	1 (1.5)	0 (0)	1 (3)	N.A.
	Hemiplegia, No. (%)	4 (5.9)	3 (8.6)	1 (3)	0.331
	Peptic ulcer disease, No. (%)	3 (4.4)	2 (5.7)	1 (3)	0.59
	Connective tissue disease, No. (%)	4 (5.9)	2 (5.7)	2 (6.1)	0.951
	Solid tumor, No. (%)	40 (58.8)	21 (60)	19 (57.6)	0.839
	Leukemia, No. (%)	2 (2.9)	0 (0)	2 (6.1)	N.A.
	Lymphoma, No. (%)	3 (4.4)	2 (5.7)	1 (3)	0.59
	Vasopressors, No. (%)	15 (22.1)	4 (11.4)	11 (33.3)	0.029
	Acute renal failure, No. (%)	9 (13.2)	4 (11.4)	5 (15.2)	0.65
	Infective endocarditis, No. (%)	5 (7.2)	0 (0.0)	4 (12.1)	N.A.
Clinical status at positive culture					
	Intensive care unit admission, No. (%)	23 (33.8)	8 (22.9)	15 (45.5)	0.049
	Total parenteral nutrition, No. (%)	10 (14.7)	3 (8.6)	7 (21.2)	0.141
	Prior surgery, No. (%)	16 (23.5)	7 (20)	9 (27.3)	0.479
	Severe sepsis, No. (%)	22 (32.4)	7 (20)	15 (45.5)	0.024
	Immunosuppressive therapy, No. (%)	7 (10.3)	5 (14.3)	2 (6.1)	0.264
	Neutropenia, No. (%)	6 (8.8)	2 (5.7)	4 (12.1)	0.351
	CVC use, No. (%)	42 (61.8)	16 (45.7)	26 (78.8)	0.005
	Early CVC removal, No. (%)	22 (32.4)	9 (56.3)	13 (50)	0.693
	CVC duration, days, median (IQR)	3 (2–5.75)	3 (2–6.75)	3 (2–5.75)	0.839
	Urine catheter, No. (%)	8 (11.8)	2 (5.7)	6 (18.2)	0.11
	Concommittant bacteremia, No. (%)	21 (30.9)	8 (22.9)	13 (39.4)	0.14
Prognosis					
	30-day mortality, Death (%)	12 (17.6)	6 (17.1)	6 (18.2)	0.91
Antifungal therapy					
	Any antifungal agent use, No. (%)	57 (83.8)	27 (77.1)	30 (90.9)	0.123
	Azoles	37 (54.4)	18 (51.4)	19 (57.6)	0.61
	Echinocandins	41 (60.3)	17 (48.6)	24 (72.7)	0.041
	Amphotericin B	3 (4.4)	0 (0)	3 (9.1)	N.A.

Abbreviations: CVC, central venous catheter; N.A., not available.

## Data Availability

The original contributions presented in the study are included in the article, further inquiries can be directed to the corresponding author.
